# Yeast Nucleolin Nsr1 Impedes Replication and Elevates Genome Instability at an Actively Transcribed Guanine-Rich G4 DNA-Forming Sequence

**DOI:** 10.1534/genetics.120.303736

**Published:** 2020-10-26

**Authors:** Shivani Singh, Alexandra Berroyer, Minseon Kim, Nayun Kim

**Affiliations:** * Department of Microbiology and Molecular Genetics, University of Texas Health Science Center at Houston, Houston, Texas 77030; † University of Texas (UT) Health MD Anderson Cancer Center Graduate School of Biomedical Sciences, Houston, Texas 77030

**Keywords:** G4 DNA, genome instability, nucleolin, replication

## Abstract

A significant increase in genome instability is associated with the conformational shift of a guanine-run-containing DNA strand into the four-stranded G-quadruplex (G4) DNA. The mechanism underlying the recombination and genome rearrangements following the formation of G4 DNA *in vivo* has been difficult to elucidate but has become better clarified by the identification and functional characterization of several key G4 DNA-binding proteins. Mammalian nucleolin (NCL) is a highly specific G4 DNA-binding protein with a well-defined role in the transcriptional regulation of genes with associated G4 DNA-forming sequence motifs at their promoters. The consequence of the *in vivo* interaction between G4 DNA and nucleolin in respect to the genome instability has not been previously investigated. We show here that the yeast nucleolin Nsr1 is enriched at a G4 DNA-forming sequence *in vivo* and is a major factor in inducing the genome instability associated with the cotranscriptionally formed G4 DNA in the yeast genome. We also show that Nsr1 results in impeding replication past such a G4 DNA-forming sequence. The G4-associated genome instability and the G4 DNA-binding *in vivo* require the arginine-glycine-glycine (RGG) repeats located at the C-terminus of the Nsr1 protein. Nsr1 with the deletion of RGG domain supports normal cell growth and is sufficient for its pre-rRNA processing function. However, the truncation of the RGG domain of Nsr1 significantly weakens its interaction with G4 DNA *in vivo* and restores unhindered replication, overall resulting in a sharp reduction in the genome instability associated with a guanine-rich G4 DNA-forming sequence. Our data suggest that the interaction between Nsr1 with the intact RGG repeats and G4 DNA impairs genome stability by precluding the access of G4-resolving proteins and impeding replication.

G-QUADRUPLEXES or G4 DNAs are unique noncanonical four-stranded DNA structures that can form from guanine-rich nucleic acid sequences (Bochman *et al.* 2012; Maizels and Gray 2013). Four guanine molecules interact via Hoogsteen bonds to yield G-quartets that in turn stack on top of each other, held together by intervening loops of variable length and sequence to form the G4 DNA. The size and orientation of the loops can determine the relative stability of various G4 DNA configurations (Kim 2019). Though some computational analyses revealed that >375,000 and >1,400 G4 motifs are in the human and the *Saccharomyces cerevisiae* genomes, respectively, it is still unclear how many of these sequences with high quadruplex-forming potential actually assume the stable G4 configuration *in vivo* (Huppert and Balasubramanian 2005; Capra *et al.* 2010). Nevertheless, it is becoming quite evident that these structural transformations potentially provide a substantial informational capacity to DNA with specific functions (Maizels and Gray 2013). Recent bioinformatic studies in human, yeast, and bacterial genomes have revealed that G4 motifs are not randomly located within genomes but are particularly enriched in certain functional regions, such as those in ribosomal and telomeric DNA, G-rich micro- and minisatellites, and mitotic and meiotic double-strand break (DSB) sites (Todd *et al.* 2005; Rawal *et al.* 2006; Eddy and Maizels 2008). Their high prevalence in and around genes particularly proximal to transcriptional start sites suggests a possible role as *cis*-acting regulatory elements (Du *et al.* 2008; Hershman *et al.* 2008; Moruno-Manchon *et al.* 2017).

The presence of G4 DNA was recently shown to impair DNA replication by hindering the progress of replicative polymerases, and the failure to resolve these structures transforms the sequence motifs into potential hotspots for genomic instability (Sarkies *et al.* 2010). In humans, the occurrence of G4 DNA motifs reportedly overlap with recombination-prone regions such as certain proto-oncogenes and the sites of frequent translocation breakpoints (Siddiqui-Jain *et al.* 2002; Seenisamy *et al.* 2004). Consistently, chromosomal translocations in the proximity of G4 motifs have been observed in leukemias and lymphomas (Bacolla *et al.* 2016). The potential to adopt G4 DNA has additionally been correlated with a number of human neurological diseases, such as frontotemporal dementia (FTD), amyotrophic lateral sclerosis, Alzheimer’s and fragile X syndrome (Majounie *et al.* 2012; Haeusler *et al.* 2014; Maizels 2015). The resolution of G4 DNA structures is thus imperative in preserving genome integrity.

The genetic tractability of *S. cerevisiae* has provided considerable insight into the mechanisms involved in maintaining stability of G-rich repetitive sequences. The instability at the G-rich human minisatellite CEB1 inserted in the *S. cerevisiae* genome was shown to be dependent on the ability of the CEB1 motif to form G4 DNA and was not observed with other tandem repeats lacking G4 DNA-forming potential (Lopes *et al.* 2011; Piazza *et al.* 2012). The G4-forming sequence derived from the guanine-run containing immunoglobulin switch Mu (Sμ) region becomes highly unstable when actively transcribed in the context of the yeast genome. Transcription conferred a critical strand bias, since genome rearrangements at Sµ were elevated only when the guanine-runs were located on the nontranscribed strand (Kim and Jinks-Robertson 2011). The direction of replication and transcription, when in a head-on orientation, further elevated genome instability at the Sµ sequence (Yadav *et al.* 2014). At the Sµ sequence, the lack of functional topoisomerase 1 (Top1) significantly elevated various types of genome instability, likely by facilitating the structural transition of a G-rich sequence to a G4 structure due to the accumulation of negative helical stress in DNA. The loss of heterozygosity and copy number alterations (deletions and duplications), both of which are frequently observed in cancer genomes, were also elevated when the Sµ sequence was actively transcribed.

The biological functions of G4 DNA are largely dependent on the protein factors that modulate the G4-conformation and/or serve as a bridge to recruit additional protein regulators (Brázda *et al.* 2014). These G4-binding proteins can be classified into three functional groups: (1) telomere-related proteins, such as the shelterin complex, human CST (CTC1-STN1-TEN1), and yeast Rap1 and Est1 (Pedroso *et al.* 2009; Li *et al.* 2013; Bhattacharjee *et al.* 2017); (2) proteins that unfold and/or process the G4 structure, such as the helicases including RecQ family helicases hBLM, hWRN, ySgs1, and yPif1 (Mendoza *et al.* 2016); and (3) proteins that stabilize G4 structures including MAZ and nucleophosmin (Gallo *et al.* 2012; Cogoi *et al.* 2014). Mutations in some of these G4-interacting proteins have been linked to genetic diseases such as Werner syndrome, Fanconi anemia, and cancer (Cantor *et al.* 2001; Seal *et al.* 2006; Wu *et al.* 2008; Mendoza *et al.* 2016). A defective BLM helicase failed to unwind G4 DNA and caused increasing recombination frequencies and a high incidence of cancer in Bloom’s syndrome (Sun *et al.* 1998). More recently, the cotranscriptional activator Sub1, which interacts with both G4 DNA and the G4-helicase Pif1, was shown to suppress the G4-associated genome instability by facilitating the recruitment of Pif1 helicase to cotranscriptionally formed G4 DNA structures (Lopez *et al.* 2017).

The human nucleolin (NCL) is a highly abundant and conserved nucleolar phosphoprotein. Its major function is in ribosomal RNA maturation with additional roles in chromatin remodeling, transcription, and apoptosis (Tajrishi *et al.* 2011). The altered expression and subcellular localization of NCL is a common biomarker of a variety of cancers demonstrating its clinical relevance (Otake *et al.* 2007; Berger *et al.* 2015; Satake *et al.* 2018; Kim 2019). Although initially described as a G4 RNA-binding protein, more recent evidence indicates that NCL preferentially and selectively binds both endogenous and exogenous G-rich sequences that can fold into G4 DNA (Dempsey *et al.* 1999; Hanakahi *et al.* 1999; Fry 2007). It is suggested that NCL acts as a chaperone to promote the correct folding of complex nucleic acid structures (González *et al.* 2009; González and Hurley 2010; Tosoni *et al.* 2015). Together with the nuclear riboprotein hnRNPD, NCL forms a lymphocyte-specific complex LR1 (lipopolysaccharide responsive factor 1), which binds at the G4 DNA-forming Immunoglobulin heavy chain (IgH) switch regions (Dempsey *et al.* 1999). The binding of NCL to the G4-forming hexanucleotide repeat expansion (HRE) (GGGGCC)_n_ in C9orf72 has been reported to be responsible for the initiation of molecular cascades that lead to neurodegenerative diseases (Haeusler *et al.* 2014). NCL is composed of three main structural domains; the amino-terminal domain containing four acidic stretches was shown to induce chromatin decondensation through interaction with histone 1H (Erard *et al.* 1988), while the central region containing tandem RNA-binding domains (RBDs) and the multiple RGG (arginine/glycine/glycine) boxes at the C-terminal domain contribute to its high-affinity interaction with G4 DNA (Hanakahi *et al.* 1999; Ghosh and Singh 2018).

Similar to the human protein, the yeast nucleolin Nsr1 has been demonstrated to be involved in pre-rRNA processing and ribosome production (Kondo and Inouye 1992; Lee *et al.* 1992). While there are no significant sequence similarities in the amino-terminal regions between yNsr1 and hNCL, the C-terminal half of yNsr1, consisting of two tandemly repeated putative RBDs and the multiple RGG (arginine/glycine/glycine) motifs, has a high sequence similarity to the carboxyl-terminal part of hNCL (37% identity in 249 amino acids) (Lee *et al.* 1991; Kondo and Inouye 1992). Unlike the plethora of studies highlighting the NCL/G4 interaction, the role of yeast Nsr1 related to G4 DNA has not been extensively studied. In the current study, we examined whether the yeast Nsr1 has a role in the genome instability associated with G4-forming sequences. We identified an important biological role of Nsr1 in enhancing various types of genome rearrangements associated with cotranscriptionally formed G4 DNA. We show that the yeast Nsr1 is enriched at the highly transcribed G4 DNA-forming motif *in vivo* and that the disruption of Nsr1 lowers the G4-associated recombination, while its overexpression further exacerbates instability in a dose-dependent manner in cells lacking Top1. The C-terminal RGG domain of Nsr1 is required to form a complex with G4 DNA *in vivo*, to obstruct replication, and to promote genetic instability, but is dispensable for the rRNA processing function of Nsr1. Our results point to an important role of Nsr1 in G4-associated genome maintenance.

## Materials and Methods

### Yeast strains and plasmids

Yeast strains used for the mutation and recombination assays were derived from YPH45 (*MATa*, *ura3-52 ade2-101 trp1Δ1*). Construction of strains containing the *pTET-lys2-GTOP* or *–GBTM* constructs were previously described (Kim and Jinks-Robertson 2011). Gene deletions were carried out via the one-step allele replacement by amplification of loxP-flanked marker cassettes. Nsr1-expression plasmid was constructed by amplifying NSR1 ORF along with 490 nt upstream and 250 nt downstream from the yeast genomic DNA and cloning into the yeast *CEN* vector pRS316. The deletion constructs N-Term Nsr1 and C-term Nsr1 have been previously described (Azevedo *et al.* 2015). The expression plasmid for human nucleolin was constructed by amplifying the NCL ORF from GFP-nucleolin (#28176; Addgene) using primers NCL-For and NCL-1XHA Rev (Supplemental Material, Table S1). The BamHI/XhoI-digested NCL PCR product was cloned into BamHI/XhoI-digested pGPD2 (#43972; Addgene). The NCL-∆RGG plasmid was similarly created using primers NCL-For and NCL∆RGG -1XHA Rev (Table S1).

### Determination of rates

Recombination rates and 95% confidence intervals were determined using the method of the median as described previously (Spell and Jinks-Robertson 2004). Twelve to 36 individual cultures were used to determine each rate and the associated 95% confidence intervals. Recombination rates are considered to be statistically different when the 95% confidence intervals, which are indicated in each graph as error bars, do not overlap. For the gross chromosomal rearrangement (GCR) assay, 5-ml cultures in YPD medium (1% yeast extract, 2% Bacto-peptone, 2% dextrose, and 250 µg/ml adenine hemisulfate) were inoculated with single colonies and grown for 3 days at 30°. Cells were then plated either on YPD-agar or synthetic complete dextrose medium lacking arginine (SCD-arg) and containing canavanine (60 mg/liter) and 5-fluoroorotic acid (5-FOA; 1 g/liter) (Chen and Kolodner 1999).

### Growth curve

Growth curve and doubling time measurement: eight independent cultures of each genotype were grown to midlogarithmic phase, diluted to ∼1 × 10^6^ cells/ml and incubated in a 96-well plate at 30°. OD_600_ was measured automatically every 10 min. Doubling time was calculated using the following equation: Doubling time = [hours cells grown Ln(2)]/[Ln (Nt/N0)], where Nt and N0 are the OD_600_ at two different times in log phase of growth.

### Northern blot analysis

Northern blot analysis of the ribosomal RNAs was carried out as previously described (Han and van Hoof 2016).

### Chromatin immunoprecipitation

For Chromatin IP (ChIP), a previously described protocol was used (Lopez *et al.* 2017) with the following modifications. Anti-FLAG antibody-conjugated beads (Cat# A8592; Sigma, were used in pull-down. Ct values for each ChIP samples were first normalized to the corresponding input samples, and then divided by the values for the *CAN1* locus to calculate the relative fold enrichment. Primers used in the qPCR analysis were previously described (Lopez *et al.* 2017). *P*-values were calculated using the Student *t*-test. For the TMPyP4 ChIP, the cells were grown at 30° in liquid YPD containing 50 µM TmPyP4. Next day, they were diluted in liquid YPD containing 50 µM TmPyP4 and grown till mid-log phase (O.D_600_ 0.7–0.8). Cells were cross-linked and further processed as above.

### Cell synchronization and time course experiment

Cell synchronization and sample collection was carried out as described with slight modifications (Owiti *et al.* 2018). Briefly, 30° grown, log phase cells (OD_600_ ∼ 0.5–0.6) with *bar1∆* were arrested in late G1 phase using 50 ng/ml α-factor peptide (Sigma) for ∼120 min, released by washing with fresh YPD medium with 50 µg/ml Pronase (Sigma), and then allowed to proceed through a synchronous cell cycle at 21°. Samples were removed at 10-min intervals and immediately treated with buffer containing 0.1% sodium azide.

### DNA extraction and ddPCR

Yeast genomic DNA extraction from the cells collected at 10-min intervals after the release from α-factor was carried out as described (Batrakou *et al.* 2018). DNA concentration was measured using the Qubit double-stranded DNA (dsDNA) HS assay (ThermoFisher). The DNA was sonicated for two cycles of 15 sec ON/90 sec OFF in Bioruptor (Diagenode) at 4°. The ddPCR reaction consisted of 1.5–2 ng of sonicated genomic DNA, 1× QX200 ddPCR EvaGreen Supermix (Bio-Rad) and primers (200 nM final each). The samples were processed using the QX200 Droplet Digital PCR system and analyzed with the QuantaSoft software (Bio-Rad). The primers are listed in Table S1. For calculating the copy number, each time point values of the “ARS306,” “KanMX,” and “STE50” loci were normalized to time 0 value of the “ARS306” locus, and for the “KanMX” and “STE50” loci, they were further normalized to the “KanMX” (time 0) and “STE50” (time 0), respectively. Data from at least three independent experiments were used to calculate the standard deviation. *P*-values were calculated using Student *t*-test (Graphpad Prism).

### Data availability

The authors state that all data necessary for confirming the conclusions presented in the manuscript are represented fully within the manuscript. Supplemental material available at figshare: https://doi.org/10.25386/genetics.13135823.

## Results

### Nsr1 enhances G4-mediated recombination in cells lacking topoisomerase 1

To understand the elevated genome instability associated with G4-forming sequences, we previously developed a recombination reporter assay in the yeast model system (Kim and Jinks-Robertson 2011). In this reporter assay, a model G4-forming sequence from the murine immunoglobulin heavy chain switch Mu region (Sμ) (Figure S1A) was inserted into the yeast genome within the LYS2 gene under the control of a tetracycline/doxycycline-repressible promoter (pTET). The Sμ sequence was inserted either in the physiological (GTOP) or into the inverted orientation (GBTM), placing the G-runs in the nontranscribed strand (NTS) or in the transcribed strand (TS), respectively (Figure S1B). The formation of G4 DNA is favored when the G-rich strand located on the NTS is transformed into a single-stranded state during transcription, freeing the guanine bases to interact with each other through Hoogsteen base-pairing. When G-runs are located in the TS, they will be occupied in base-pairing with the nascent RNA strand and will not be free to fold into G4 DNA. Thus, any factors involved in the formation or stability of G4 DNA should affect the recombination occurring at the *pTET-lys2-GTOP* construct, with little to no effect on the rate of recombination at the *pTET-lys2-GBTM* construct (Kim and Jinks-Robertson 2011; Yadav *et al.* 2014; Lopez *et al.* 2017).

**Figure 1:**
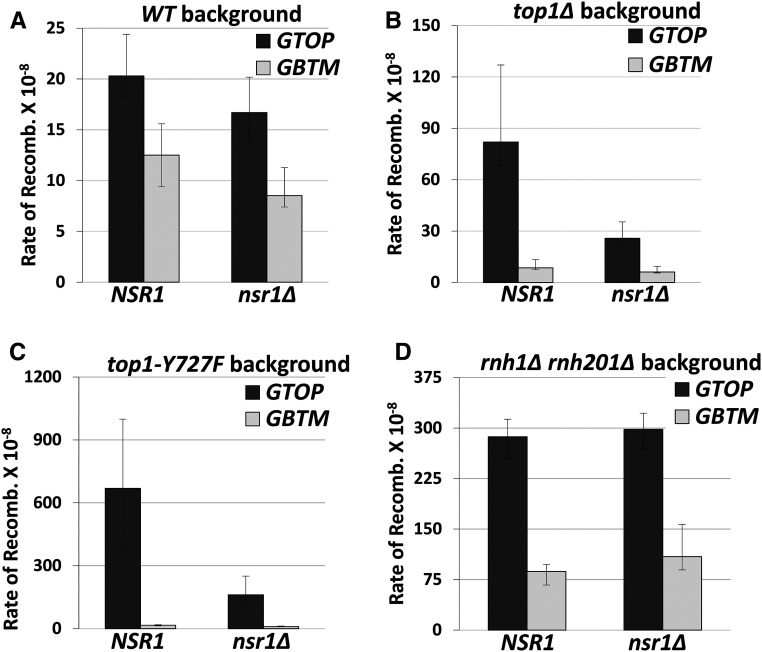
The rates of G4-associated recombination in *nsr1∆* strains. Guanine-runs are on the nontranscribed, top strand in a single-stranded state in *pTET-lys2-GTOP* cassette and on the transcribed strand annealed to the nascent RNA in the pTET-lys2–GBTM cassette. All graphs show the rates of recombination (×10^−8^). Error bars indicate 95% confidence intervals. Two rates are considered statistically different when the confidence intervals do not overlap. The rates, numbers of cultures used in fluctuation analyses, and 95% confidence intervals are listed in Table S2. (A) Recombination rates in WT backgrounds. (B) Recombination rates in *top1∆* backgrounds. (C) Recombination rates in *top1Y727F* backgrounds. (D) Recombination rates in *rnh1∆ rnh201∆* backgrounds.

To determine whether Nsr1 plays a role in the G4-mediated genome instability, we deleted the *NSR1* gene in strains containing the *pTET-lys2-GTOP* or -GBTM construct and checked the rate of recombination at this locus. In the wild-type (WT) background, the deletion of *NSR1* resulted in a slow-growth phenotype of the cells as previously reported (Lee *et al.* 1992). Under high transcription conditions, the rates of recombination at the *pTET-lys2-GTOP* or *-GBTM* cassette in *nsr1∆* strains were not changed from those in *WT* strains (Figure 1A).

Topoisomerase I (Top1) was previously identified to be a crucial factor for G4-mediated genome instability in yeast (Kim and Jinks-Robertson 2011; Yadav *et al.* 2014; Yadav *et al.* 2016). Accumulation of negative supercoils in *top1Δ* yeast cells was shown to promote recombination at the *pTET-lys2-GTOP* construct. To determine whether Nsr1 modulates the G4-associated genomic instability in the absence of Top1, we deleted the *NSR1* gene in a *top1Δ* background. The rate of recombination for the *pTET-lys2-GTOP* was reduced by ∼threefold in the t*op1Δ nsr1Δ* strain compared to *top1Δ* (Figure 1B). For the *pTET-lys2-GBTM*, the rates of recombination were indistinguishable between *top1Δ* and *top1Δ nsr1Δ* backgrounds. When the transcription from the *pTET* promoter was repressed by the addition of 2 μg/ml doxycycline in the media, the deletion of *NSR1* did not affect the rates of recombination in WT and reduced slightly in a *top1∆* background (Figure S2).

The catalytically inactive mutant of Top1 (Top1-Y727F) with the mutation of catalytic tyrosine to phenylalanine results in complete ablation of its function in removal of supercoils, while not affecting its DNA-binding activity (Megonigal *et al.* 1997). Possibly due to its high G4-binding ability, the expression of Top1-Y727F results in recombination rates that are ∼eightfold higher than in the absence of Top1 (*top1∆*) (Yadav *et al.* 2016). When Nsr1 was disrupted in the *top1Y727F* backgrounds, the recombination rate at the *pTET-lys2-GTOP* cassette was significantly decreased and was comparable to the rate in the *top1∆* strain (Figure 1C). The disruption of Nsr1 did not affect the rate of recombination at the *pTET-lys2-GBTM* cassette.

### Nsr1 does not affect the G4-associated recombination in RNase H-deficient cells

Disrupting both RNase H1 and RNase H2 in yeast leads to the accumulation of transcription-associated RNA:DNA hybrids or R-loops (Wahba *et al.* 2011) and subsequently elevates levels of recombination for both the *pTET-lys2-GTOP* and -GBTM constructs (Kim and Jinks-Robertson 2011). The G-loop, a higher order structure consisting of R-loop and G4 DNA, could arise from either the enhanced negative supercoils leading to G4 formation upon disruption of Top1 or due to the failure to degrade RNA leading to R-loops upon disruption of RNase Hs. We have previously shown that the elevated recombination rates for the *pTET-lys2-GTOP* and -GBTM constructs in *rnh1∆* *rnh201∆* backgrounds are reduced by either repressing transcription from *pTET* (Kim and Jinks-Robertson 2011) or by removing the RNA:DNA hybrids by ectopic expression of RNase H1 (Yadav *et al.* 2016). Therefore, we checked whether, in addition to reducing recombination rates in a *top1∆* strain, the deletion of Nsr1 could affect the R-loop-mediated enhanced recombination rates. In the triple deletion mutant *rnh1∆ rnh201∆ nsr1∆,* there was no significant alteration in the rate of recombination for the *pTET-lys2-GTOP* or -GBTM construct (Figure 1D). This data suggests that Nsr1-induced genomic instability is specific to G4 DNA formed due to the enhanced negative helical torsion under high transcription in a *top1∆* background and not due to the RNA: DNA hybrid accumulation.

### Nsr1 promotes GCRs in the *top1∆* backgrounds

**Figure 2:**
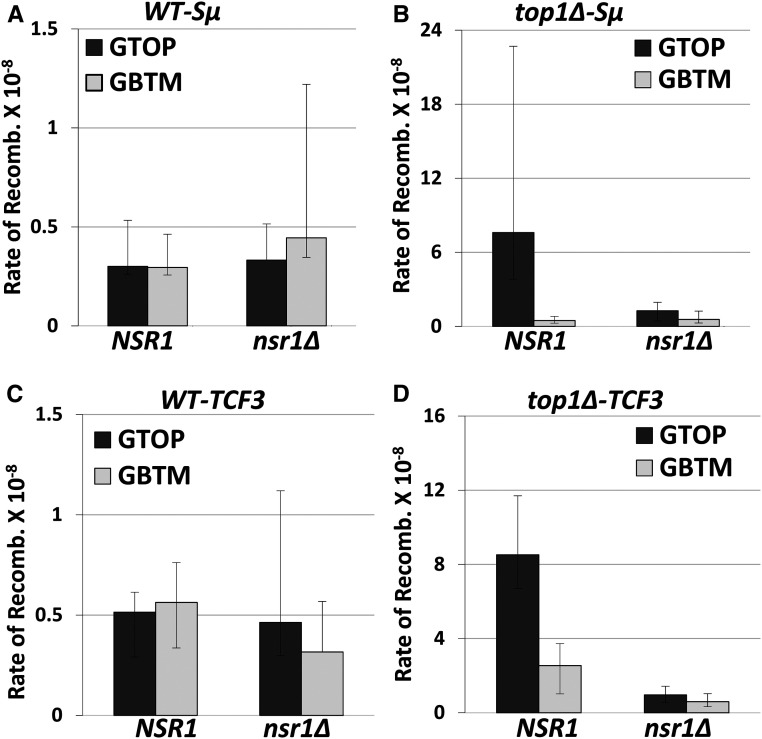
Rates of gross chromosomal rearrangement (GCR) in *nsr1∆* strains. All graphs show the rates of 5-FOA^r^/CAN^r^ or GCRs (×10^−8^). The rates of GCRs occurring at CHR5 containing the *pTET-lys2-GTOP* or -GBTM cassette are determined by the method of median. 95% Confidence intervals are indicated by the error bars. Two rates are considered statistically different when the confidence intervals do not overlap. GCR rates, numbers of cultures used in fluctuation analyses, and the 95% confidence intervals are listed in Table S4. GCR rates in strains with the reporter cassette containing the Sμ-G4 in (A) WT backgrounds and (B) *top1∆* backgrounds. GCR rates in strains with the reporter cassette containing TCF3-G4 in (C) WT backgrounds and (D) *top1∆* backgrounds.

We previously reported that, in the absence of functional Top1, the cotranscriptionally formed G4 DNA at the *pTET-lys2-GTOP* construct leads to the increase in ectopic recombination as well as in the GCRs (Yadav *et al.* 2014). To measure the rates of GCR, we used a modified form of the GCR reporter system developed by Chen and Kolodner (1999). In this reporter system, the *URA3* gene was integrated into the left arm of chromosome V (CHR5) replacing the *HXT13* gene located ∼8.5 kb centromere-distal to the *CAN1* gene. The *pTET-lys2-GTOP* or -*GBTM* cassette containing the Sμ G4 motif, as described before (Yadav *et al.* 2014), is integrated immediately centromere-proximal to *CAN1*. The loss of functional CAN1 or *URA3* results in resistance to the drug canavanine (Can) or 5-fluoroorotic acid (5-FOA), respectively. Thus, by simultaneous selection against two counterselectable markers (URA3 and CAN1), complex genome rearrangement can be measured. Using this modified GCR assay, it was previously shown that in the high transcription conditions, the disruption of Top1 leads to a significantly higher (∼30-fold) GCR rate for *pTET-lys2–GTOP*, where guanine-runs are present on the NTS, compared to the *pTET-lys2–GBTM* construct where guanine-runs are on the TS (Yadav *et al.* 2014). When we deleted NSR1 in WT backgrounds, there was no change in the GCR rates for *pTET-lys2–GTOP* or *-GBTM* (Figure 2A). However, the disruption of Nsr1 in *top1Δ* backgrounds resulted in a significant, ∼sixfold reduction of the GCR rates for the *pTET-lys2-GTOP* but not for the *-GBTM* construct (Figure 2B). To test whether the function of Nsr1 is specific to the Sμ G4-mediated genome instability or extends to other G4 motifs as well, we used a GCR reporter modified to contain the G4 motif from the TCF3 translocation breakpoint (Williams *et al.* 2015). In a similar manner to the GCR reporter containing Sµ G4, when Nsr1 was disrupted in a *top1Δ* strain but not in a WT strain, the GCR rates were severely lowered by ∼ninefold at the *pTET-lys2-GTOP (TCF3)* construct, while at the *pTET-lys2-GBTM (TCF3)* construct the GCR rates were not significantly changed (Figure 2, C and D).

### The RGG domain of Nsr1 is necessary for the elevated G4-associated recombination but not for the support of normal cell growth or pre-rRNA processing

We considered the possibility that the effect of *NSR1* deletion on the rate of recombination could be attributed to the marked slow-growth phenotype reported for *nsr1∆* cells (Kondo and Inouye 1992; Lee *et al.* 1992). We measured cell growth and calculated doubling time in WT, *top1∆*, *nsr1∆*, and *top1Δ nsr1Δ* strains. While the doubling time of a WT (∼96 min) and *top1Δ* strain (∼99 min) was comparable, the *nsr1Δ* mutant strain showed a significantly longer doubling time of 141 min (Figure S3A). This growth defect was slightly further aggravated upon the deletion of the *TOP1* gene; the doubling time was ∼152 min for the *top1Δ nsr1Δ* mutants. Nsr1 has been reported to be involved in pre-rRNA processing in yeast, and deletion of the *NSR1* gene leads to defective 35S pre-rRNA processing; 35S accumulates and 20S is greatly reduced (Lee *et al.* 1992). We carried out Northern hybridization analysis using oligonucleotide probes that were designed to hybridize to pre-rRNA and confirmed the sharp decrease in the 20S and the concomitant accumulation of unprocessed 35S in our *nsr1Δ* strain (Figure S3, B and C). The deletion of the *TOP1* gene had no or little effect on the pre-rRNA processing in WT or *nsr1∆* backgrounds, respectively. The C-terminal RGG domain of nucleolin has been shown to be important for inducing and stably binding G4 structures (González and Hurley 2010; Ghosh and Singh 2018). To test whether the deletion of the RGG domain results in a cell growth defect similar to the complete null allele, we replaced the WT *NSR1* allele with the *nsr1∆RGG* allele at its endogenous chromosomal location on the left arm of chromosome 7 in a *top1∆* background. In this mutant strain (*top1∆ nsr1∆RGG*), a growth defect was not observed, and the doubling time was similar to the WT or *top1∆* strain rather than *nsr1∆* or *top1∆ nsr1∆*. The pre-rRNA processing efficiency in the *top1Δ nsr1ΔRGG* strain was also similar to that of the WT or *top1∆* strain with a similar ratio of 20S/35S. We also observed no differences in 7S pre-rRNA level within the different mutants (Figure S3B). Thus, the RGG domain appears dispensable for the normal cell growth and pre-rRNA processing function.

**Figure 3:**
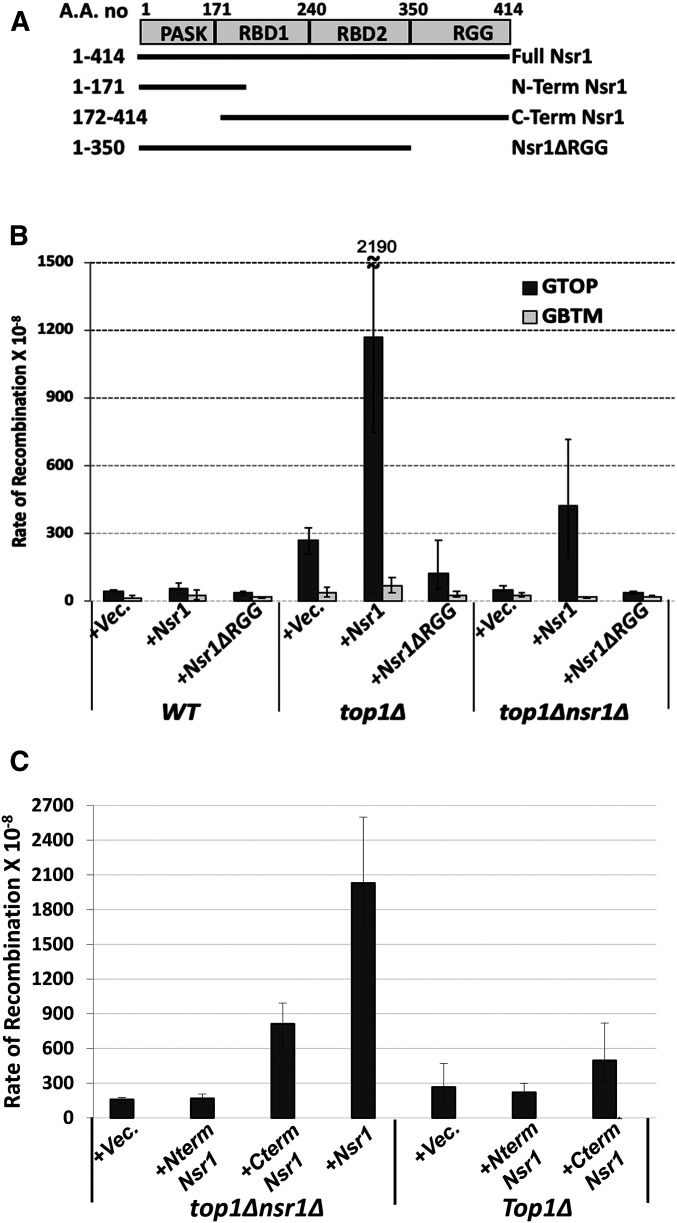
Complementation of NSR1-deletion. (A) Domain organization of Nsr1. The deletion constructs used for complementation are indicated. (B and C) Graphs show the rates of recombination (×10^−8^). Error bars indicate 95% confidence intervals. Two rates are considered statistically different when the confidence intervals do not overlap. For B, indicated yeast strains are transformed with a *CEN* plasmid [an empty vector (Vec.) or a plasmid with either pNSR1-Nsr1 or pNSR1-Nsr1∆RGG construct]. The rates, numbers of cultures used in fluctuation analyses, and 95% confidence intervals are listed in Table S5. For C, indicated yeast strains are transformed with a *2µ* plasmid [an empty vector (Vec.) or a plasmid with either pADH1-Nsr1, pADH1-Nterm Nsr1, or pADH1-Cterm Nsr1 construct]. The rates, numbers of cultures used in fluctuation analyses, and 95% confidence intervals are listed in Table S6.

To further test whether Nsr1 or Nsr1∆RGG can elevate the recombination rate at the *pTET-lys2-GTOP*, a full-length *NSR1* gene and the N-terminal 1–350 residues of Nsr1 lacking the RGG domain (*nsr1ΔRGG)* along with its own promoter (*pNSR1*) were each cloned into the centromeric plasmid pRS316 (Figure 3A). The ectopic expression of full-length Nsr1 in the *top1Δ nsr1Δ* double mutant strain elevated the rate of recombination for the *pTET-lys2-GTOP* by 8.5-fold, thereby resulting in the rates of recombination that were similar to a *top1Δ* transformed with the empty vector (Figure 3B). However, the expression of Nsr1*∆*RGG did not elevate the rate of recombination at the *pTET-lys2-GTOP* cassette. The rates of recombination of the *pTET-lys2-GBTM* cassette were not affected by either Nsr1-or Nsr1ΔRGG expression. This result, which indicates that the RGG domain is necessary to elevate recombination at the *pTET-lys2-GTOP* cassette, was confirmed using the yeast strains where the WT *NSR1* allele was replaced with the *nsr1∆RGG* allele at its endogenous chromosomal location on the left arm of chromosome 7. Similar to the results obtained with plasmid-expressed Nsr1ΔRGG, the deletion of the RGG domain (*nsr1∆RGG*) at its genomic location resulted in the reduced recombination rate at the *pTET-lys2-GTOP* cassette similar to the complete deletion (*nsr1∆*) (Figure S3D). Together, these results indicate that even though Nsr1∆RGG was sufficient to support normal yeast cell growth and rRNA processing functions (Figure S3, A–C), it is necessary for elevated genome instability at the G4-forming *pTET-lys2-GTOP* cassette.

We next expressed the N-terminal (1–171aa; N-term Nsr1 in Figure 3A) or the C-terminal region (C-Term; 172–414aa) of Nsr1 using 2µ plasmid constructs with the highly expressed *ADH1* promoter. The N-term construct was unable to complement for the loss of Nsr1 in enhancing G4-induced recombination (Figure 3C). However, the C-terminal deletion construct significantly elevated the rates of recombination albeit not as robustly as the full-length Nsr1. The rates of recombination for the C-terminal deletion construct were 5.1-fold higher than the rates of recombination for the empty vector. Although the effect of the C-terminal construct (*2µ* plasmid with *ADH1* promoter) cannot be directly compared to the effect of Nsr1∆RGG (*CEN* plasmid with *NSR1* promoter), these results overall suggest that the RBD and RGG domains at the C-terminal of Nsr1 are required for promoting G4-associated instability.

When we expressed the full-length Nsr1 and Nsr1ΔRGG in WT strains with the normal endogenous level of Nsr1, there was no effect of overexpression of the full-length Nsr1 constructs, while the Nsr1ΔRGG construct reduced the rates of recombination slightly (Figure 3B). In the *top1∆* strain, the overexpression of full-length Nsr1 elevated the rates of recombination at the *pTET-lys2-GTOP* cassette by ∼4.5-fold compared to vector alone. When the Nsr1*Δ*RGG construct was expressed in the *top1∆* strain, the rates of recombination were consistently lower than the empty vector. The N-term Nsr1 (1–171) was unable to induce recombination and showed rates of recombination that were similar to the empty vector. The C-terminal Nsr1 (172–414) expression resulted in about twofold higher recombination rates (Figure 3C). These results suggest that Nsr1 increases recombination in a dose-dependent manner, and this function requires the RGG domain.

**Figure 4:**
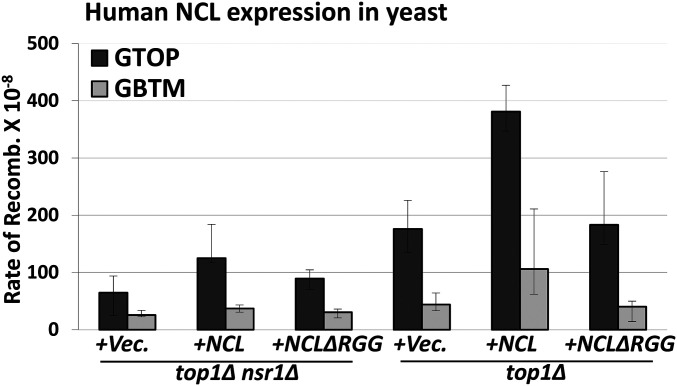
Overexpression of Nsr1 or hNCL. The *top1∆ nsr1∆* or *top1∆* yeast cells were transformed with empty vector (+Vec), hNCL-expression plasmid (+NCL) and hNCL-∆RGG followed by fluctuation analysis to determine the rates of recombination. All graphs show the rates of recombination (×10^−8^). Error bars indicate 95% confidence intervals. Two rates are considered statistically different when the confidence intervals do not overlap. The rates, numbers of cultures used in fluctuation analyses, and 95% confidence intervals are listed in Table S7.

We also tested whether the highly conserved human NCL can complement the loss of Nsr1 in yeast. When NCL was expressed from a plasmid (*CEN*, *pNSR1*), the rate of recombination at the *pTET-lys2-GTOP* cassette was elevated by ∼twofold (Figure 4). And like the yeast Nsr1∆RGG, the human NCL∆RGG with C-terminal deletion failed to increase the rate of recombination in *top1∆ nsr1∆* cells. In *top1∆* cells, the expression of NCL, but not NCL∆RGG, resulted in a further twofold increase in recombination at the *pTET-lys2-GTOP*, indicating that the function of Nsr1 with an intact RGG domain in mediating the G4-associated genome instability is conserved in the human homolog (Figure 4).

### Nsr1 binds to the G4 DNA *in vivo*

**Figure 5:**
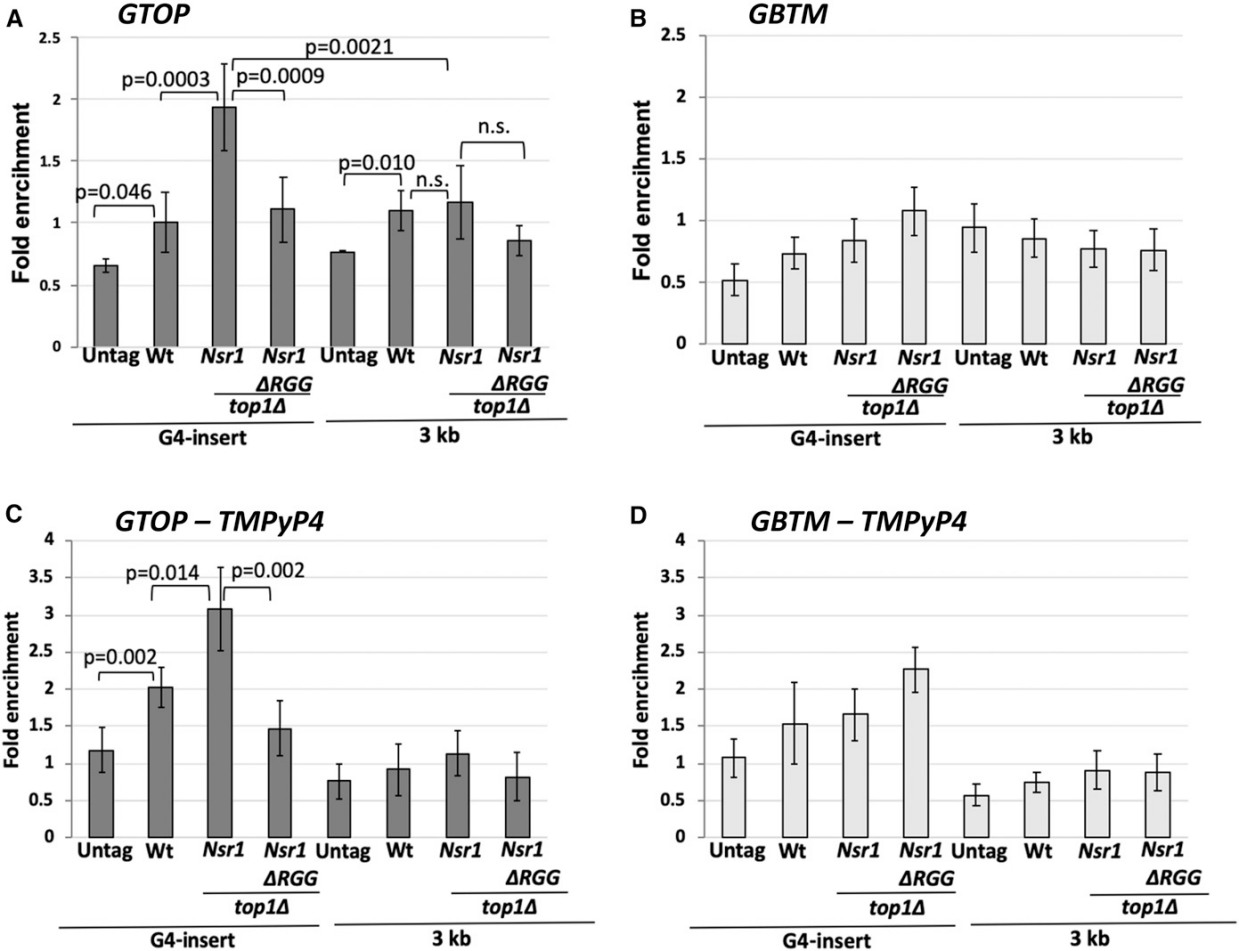
Nsr1 binding to G4 DNA *in vivo* requires the RGG domain. ChIP was carried out with an αFlag antibody and chromatin fractions prepared from yeast cells with indicated genetic backgrounds. 5¢BGL and STP primers were previously described and anneal within the LYS2 ORF at ∼100 and ∼3000 bp from the Sμ sequence insertion site, respectively. All values are based on at least six independent samples with the exception of the no-tag samples (*N* = 3) and the standard deviations are indicated by error bars. *P*-values shown were calculated using Student’s *t*-test. (A) GTOP strains; (B) GBTM strains; (C) GTOP strains treated with TMPyP4; (D) GBTM strains treated with TMPyP4.

To determine whether Nsr1 interacts with G4 structures *in vivo*, we measured the association of Nsr1 and G4 DNA by using a chromatin immuno-precipitation (ChIP) assay. ChIP was performed in WT, *top1∆*, and t*op1Δ nsr1∆RGG* cells containing either the *pTET-lys2-GTOP* or–GBTM construct and expressing Nsr1 or Nsr1∆RGG with a C-terminal 3XFlag tag. Following the pull-down with αFlag antibody-conjugated beads, qPCR analysis was done to determine the enrichment of Nsr1 at a locus 100 bp from the G4 insertion site (“G4 insert”) and a locus ∼3 kb away from the G4 insertion site (“3 kb”). We observed ∼twofold enrichment of Nsr1 at the G4 locus when the G4 sequence was in the *GTOP* orientation, but not in the *GBTM* orientation in a *top1Δ* background (*P* < 0.0001) (Figure 5, A and B). Further, the enrichment of Nsr1 was significantly higher at the switch region G4 sequence insertion site than the 3′ region of the *lys2* sequence 3 kb away (*P* = 0.0021) (Figure 5A). Nsr1∆RGG, which is missing the C-terminal RGG domain, however, was not significantly enriched at the G4 locus in *top1∆* cells (*P* = 0.0009 compared to full-length Nsr1). For the mammalian NCL, *in vitro* interaction with G4 DNA is significantly enhanced by its C-terminal domain (Hanakahi *et al.* 1999; Hanakahi *et al.* 2000; González and Hurley 2010; Tosoni *et al.* 2015). Similarly, Nsr1 forms a complex with a G4-forming oligonucleotide *in vitro*, and such interaction is diminished either by the mutation of guanine-runs involved in the quadruplex formation or by the deletion of C-terminal RGG repeats of Nsr1 protein (Figure S4). These data indicate that Nsr1 specifically associates with the G4 DNA accumulated under high transcription conditions and that this association between G4 and Nsr1 requires the C-terminal RGG repeats.

When we repeated the ChIP experiments in yeast cells treated with G4 stabilizing ligand TMPyP4 prior to cross-linking, enrichment of Nsr1 at the G4 locus when the G4 sequence was in the *GTOP* orientation was elevated by ∼threefold with no change in the enrichment at the non-G4 locus 3 kb away (Figure 5C). However, enrichment of Nsr1 was also elevated at the G4 locus when the G4 sequence was in the *GBTM* orientation (Figure 5D), possibly indicating that the guanine-runs present on the TS in the *GBTM* orientation can be induced to form relatively stable G4 DNA structure by TMPyP4.

### Nsr1-disruption allows for better access by activation-induced deaminase to the single-stranded DNA in the G4-prone sequence

Formation of a stable Nsr1–G4 DNA complex could elevate genome instability at the *pTET-lys2-GTOP* by precluding the access of G4-resolving proteins such as DNA helicases to G4 DNA. To test whether the presence of Nsr1 impedes the accessibility of protein factors to G4 DNA, we expressed human Activation-induced deaminase (hAID) in the yeast strains containing the *pTET-lys2-GTOP* or *-GBTM* construct. hAID converts cytosine to uracil and is required for the somatic hyper-mutation (SHM) of immunoglobulin variable region genes as well as the heavy chain class switch recombination (Peled *et al.* 2008). We previously showed that the ectopic expression of hAID in the WT and *top1∆* yeast cells resulted in a higher rate of recombination at the *pTET-lys2-GBTM* than *-GTOP* in both strain backgrounds (Kim and Jinks-Robertson 2011). This difference was partly attributed to the larger number of cytosines present on the single-stranded NTS in the *GBTM* orientation compared to the *GTOP* orientation (358 *vs.* 117 cytosines), since hAID specifically targets the cytosines located on the single-stranded DNA.

We postulated that the NSR1–G4 DNA complex could further impede the accessibility of hAID to those cytosines on the NTS in the *pTET*-lys2-GTOP construct located proximal to the G4 structure. Thus, we tested whether the cytosines present on the NTS in the GTOP orientation could become more accessible to hAID in the absence of Nsr1 protein. When we expressed hAID, the rate of recombination at the *pTET-lys2-GBTM* cassette was elevated by about four- to fivefold in the WT, *top1∆*, and *top1∆ nsr1∆* strains. For the *pTET*-lys2-GTOP construct, the rate of recombination was not changed by the expression of hAID in WT and *top1∆* strains, but increased by about threefold in a *top1∆ nsr1∆* strain (Table 1). This increased rate of recombination could reflect less-restricted access of hAID to the cytosines in the absence of a G4 DNA–Nsr1 complex on the NTS.

**Table 1:** Effect of hAID expression on G4-associated recombination rates

	Genotype	Recombination rate × 10^−8^ (C.I.*^a^*)		Fold change: hAID/vector
		Empty vector	hAID	
pTET-lys2-GTOP	WT	20.4 (15.1–27.9)	31.7 (16.2–60.2)	1.5
	*top1Δ*	220 (177–322)	219 (137–661)	0.99
	*top1Δnsr1Δ*	20.6 (13.7–27.8)	54.1 (46.3–65.8)	2.6
pTET-lys2-GBTM	WT	12.7 (8.0–17.1)	59.1 (44.7–84.5)	4.6
	*top1Δ*	11.4 (8.92–15.3)	45.6 (38.8–51.4)	4.0
	*top1Δnsr1Δ*	10.4 (8.3–15.5)	56.7 (29.7–150)	5.4

a 95% confidence interval.

### Nsr1 obstructs replication at a G4 DNA-containing locus

**Figure 6:**
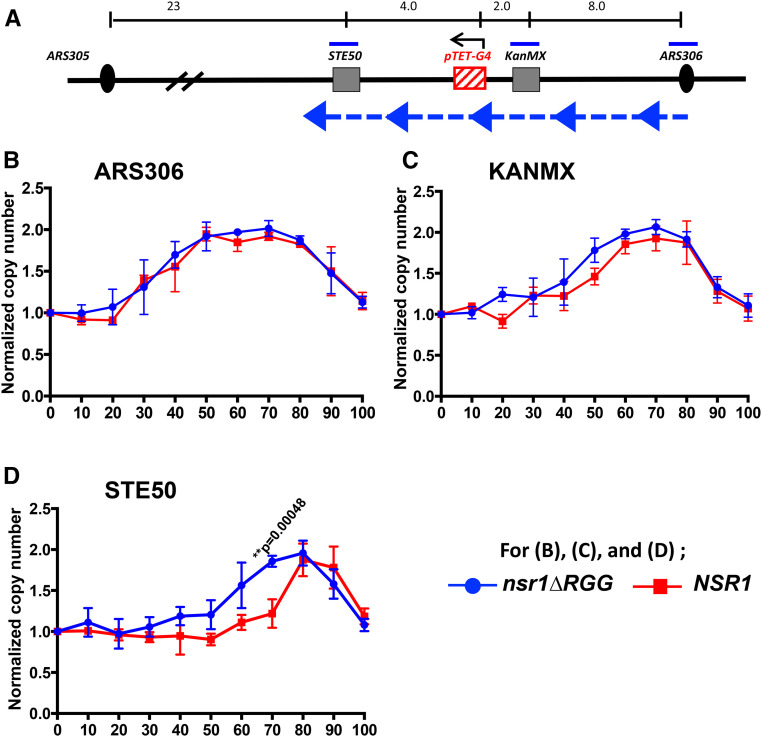
DNA replication timings and copy number changes determined by ddPCR. (A) A schematic drawing of the regions proximal to the *pTET-lys2-GTOP* cassette on yeast chromosome III. The locations of primers used in ddPCR are indicated by blue bars. The black arrow over pTET-G4 indicates the direction of transcription. Large blue arrow heads with dashed blue line indicate the direction of replication fork movement. For the sequences of the primers, see Table S1. The distance indicated above in kilobases are estimates and not to scale. (B–D) For *top∆* and *top1∆ nsr1∆RGG* strains, DNA samples analyzed by ddPCR were extracted at the indicated time points (minutes after the release from α-factor). For calculating the copy number, each time point value of “ARS306,” “KanMX”, and “STE50” loci was normalized to time 0 value of the “ARS306” locus. And for “KanMX” and “STE50” loci, they were further normalized to the “KanMX” at time 0 and “STE50” at time 0, respectively. Data from at least three independent experiments was used to calculate mean and standard deviations (indicated by error bars). *P*-values were calculated using Student’s *t*-test. All *P*-values <0.005 are indicated. Normalized copy number of (B) “ARS306”, (C) “KanMX”, and (D) “STE50” loci.

The above genetic data and enrichment of Nsr1 at the G4–insert loci led us to hypothesize that Nsr1 binds to G4 DNA *in vivo* and obstructs replication. To determine whether the Nsr1–G4 DNA complex functions as a replication block, we measured the DNA copy number of specific genomic loci throughout S phase. Droplet digital PCR (ddPCR), which provides an absolute quantification of the target DNA with high precision, accuracy, and sensitivity (Pinheiro *et al.* 2012), was used to determine the replication kinetics as inferred from the copy number changes throughout the S phase (see Materials and Methods). Our approach was modified from a previously reported use of ddPCR in determining replication timing of multiple sites in the yeast and human genomes (Batrakou *et al.* 2018). To determine the locus-specific replication timing, cells were first arrested with α-factor and released into S phase. Flow cytometry confirmed synchronous progression through S phase upon release from α-factor (Figure S5). DNA samples collected every 10 min from 0 to 100 min after the release were used to determine the copy numbers at three different loci; “ARS306” – near the early firing autonomously replicating sequence on chromosome III, “KanMX” – between “ARS306” (∼8 kb distal) and the *pTET-lys2-GTOP* cassette (∼2 kb distal), and “STE50” – further distal from “ARS306” (∼14 kb distal) compared to the *pTET-lys2-GTOP* cassette (∼4 kb distal) (Figure 6A). The other nearest origin of replication is *ARS305*, which is located >23 kb from the “STE50” locus. As marked by the blue arrows in Figure 6A, replication in this genomic region was previously determined to predominantly originate from *ARS306* and proceed past “KanMX”, *pTET-lys2-GTOP*, and then “STE50” loci (Kim *et al.* 2007). Replication kinetics at these three genomic loci in yeast cells in a *top1∆* background with either an *NSR1* or *nsr1ΔRGG* allele were compared; the significant growth defect in *nsr1∆* cells compared to *NSR1* cells, which is not present in cells with the *nsr1ΔRGG* allele (Figure S3A), would have interfered with a straightforward comparison. In respect to the recombination occurring at the *pTET-lys2-GTOP* cassette, the *nsr1ΔRGG* allele is indistinguishable from the *nsr1∆* null allele (Figure 3B and Figure S3D).

During the S phase, the copy number at each genomic locus is expected to progressively increase from one to two as replication proceeds. The time at which replication completes and the copy number reaches two will be mostly dependent on proximity to the relevant replication origin, *ARS306*. In *top1∆ nsr1∆RGG* cells containing the *pTET-lys2-GTOP* cassette, the copy number of “ARS306” locus reached two at around 50–60 min after the release from α-factor (Figure 6B). A similar pattern for this locus was observed in *top1∆* cells, which express full-length Nsr1 protein. For the *top1∆* and *top1∆ nsr1∆RGG* strains containing the *pTET-lys2-GBTM* cassette, the copy number of “ARS306” locus also reached two at around 50–60 min after the release from α-factor with no significant difference between the two strain backgrounds (Figure S6A). For the “KanMX” locus in the strains with the *pTET-lys2-GBTM* cassette, the time after release from α-factor required to reach the copy number of two was between 60 and 70 min regardless of the strain background (Figure S6B). Also, in the *top1∆* cells with the *pTET-lys2-GTOP* cassette, there was only a slight but statistically nonsignificant lag in replication at the “KanMX” locus compared to the *top1 nsr1∆RGG* cells (Figure 6C). At the “STE50” locus, there was a more significant difference in replication kinetics between the *top1∆ nsr1∆RGG* and *top1∆* strains (Figure 6D). While the copy number of this locus reached two around 70 min after the release from α-factor in *top1 nsr1∆RGG* cells, in *top1∆* cells the copy number of “STE50” was significantly below two at the 70-min time point, reaching two only 80 to ∼90 min after α-factor release.

**Figure 7:**
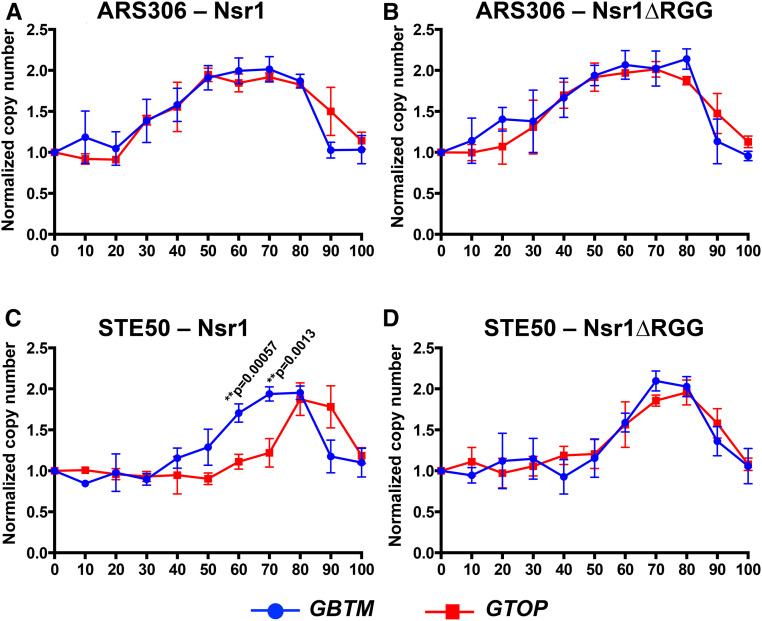
DNA replication timings near *pTET-lys2-GTOP vs. pTET-lys2-GBTM*. The DNA copy numbers determined from 0 to 100 min after the release from α-factor were plotted. The data used for the graphs are identical to those shown in Figure 6 and Figure S6. (A) “ARS306” in top1∆ background, (B) “ARS306” in top1∆ nsr1∆RGG background, (C) “STE50” in top1∆ background, and (D) “STE50” in top1∆ nsr1∆RGG background.

For *top1∆* and *top1∆ nsr1∆RGG* strains containing the *pTET-lys2-GBTM* cassette, no difference in the replication kinetics was observed with the copy number at “STE50” reaching two around 70 min after α-factor release (Figure S6C). The *pTET-lys2-GBTM* cassette contains the identical guanine-run-containing sequence from the mouse Sµ region as the *pTET-lys2-GTOP* cassette but in reverse orientation in respect to the direction of transcription within the context of the *LYS2* gene (Kim and Jinks-Robertson 2011). We have previously shown that there is no significant difference in the transcription rates of these two cassettes (Yadav *et al.* 2014). During transcription, the DNA strand containing guanine-runs is the NTS in the *pTET-lys2-GTOP* cassette and TS in the *pTET-lys2-GBTM* cassette. Although identical in sequence content, the strand difference conferred by transcription allows higher potential for the guanine-run-containing strand to assume G4 DNA due to its transient single-strandedness in the context of the *pTET-lys2-GTOP* cassette. When the replication kinetics were compared between strains containing either the *pTET-lys2-GTOP* or *pTET-lys2-GBTM* cassette, a significant difference was noted at the STE50 locus, which is replicated after the G4 DNA-forming *pTET-lys2-GTOP* or *-GBTM* cassette, only when full-length Nsr1 was present (Figure 7). Nsr1 or Nsr1∆RGG did not affect the replication kinetics at “ARS306,” which is replicated prior to the *pTET-lys2-GTOP* or *-GBTM* cassette.

## Discussion

Although initially described as a sequence-specific RNA-binding protein, human NCL preferentially binds to G-quadruplex structures over RNA substrates and plays a crucial role in G4 metabolism (González *et al.* 2009; González and Hurley 2010; Haeusler *et al.* 2014). In this study, we have explored the possibility that the yeast homolog Nsr1 also contributes to G4 DNA metabolism. We showed that, similar to the human homolog, yeast Nsr1 is significantly enriched *in vivo* at a G4 reporter construct (Figure 5). Such enrichment was only observed when the guanines are on the top, NTS of an actively transcribed gene (*i.e.*, *pTET-lys2-GTOP*) in a *top1Δ* background, which is the condition with the significant elevation of G4-associated recombination. Nsr1-enrichment is significantly reduced ∼3 kb away from the G4 motif sequence even though it is within the same transcribed unit, which further supports the specificity of Nsr1 interaction with G4 DNA.

The disruption of Nsr1 substantially decreases the G4-associated genomic instability as manifested by the decreased rates of recombination and of GCR in a *top1∆* background by about three- and sixfold, respectively (Figures 1 and 2). The reduction in genome instability in the absence of Nsr1 was not specific to the switch region G-rich sequence, as GCRs occurring at a different G-rich motif from the human TCF3 gene were also reduced upon deletion of *NSR1* (Figure 2D). Additionally, overexpression of Nsr1 led to further elevation of recombination specifically at the *pTET-lys2-GTOP* cassette in a *top1∆* background (Figure 3B). One interpretation of these surprising results is that the consequence of the association between Nsr1 and G4 DNA is the elevated genome instability. Both SµG and TCF3 fragments used in our reporter assays are typical of the recently characterized Long G4-capable regions (LG4s) that contain a series of neighboring G4-capable sequences (Williams *et al.* 2020). The function of potential multiple, closely spaced G4 DNA associated with LG4s has only been recently studied. It is therefore possible that Nsr1-dependent elevation of genome instability is limited to such LG4s rather than a singly present G4 motif.

Genome instability associated with G4-structures should be prevented by the activity of G4-unwinding DNA helicases including yeast Sgs1 and Pif1 (Huber *et al.* 2002; Paeschke *et al.* 2013). We considered the possibility that Nsr1 can occlude DNA helicases from recognizing and then resolving G4 DNA. Human nucleolin NCL, in fact, was reported to prevent Werner helicase from unwinding G4 oligos in *vitro* (Indig *et al.* 2012). When we expressed hAID to induce cytosine deamination and subsequent recombination, those cytosines present on the NTS at the G4 DNA-forming *pTET-lys2-GTOP* cassette were more susceptible to hAID processing in a *top1∆ nsr1∆* background than in a *top1∆* background (Table 1), supporting the idea that the regions proximal to the G4 DNA are in a less accessible conformation in the presence of Nsr1. These results show that Nsr1–G4 DNA complexes are capable of precluding access of DNA-interacting protein factors such as hAID to G4 DNA. We speculate that if the access of G4-specific helicase were to be precluded in a similar manner, the failure to resolve G4 DNA is expected to result in elevated genome instability.

Located C-terminal to the multiple RBDs, yeast Nsr1 and human NCL contain six and nine repeats of arginine-glycine-glycine or RGG motifs, respectively, which are found in proteins associated with important nucleic acid-mediated functions such as transcription, pre-mRNA splicing, DNA damage signaling, and mRNA translation (Thandapani *et al.* 2013). Other proteins with verified roles in DNA metabolism and cancer development that contain RGG motifs include Mre11 (a DSB-processing enzyme), Mll4 (a histone methyltransferase), and Ews (Ewings sarcoma protein; DNA damage response protein). Recently, the RGG-box of hnRNPA1, a member of ribonucleoproteins, was shown to bind specifically to the telomeric G4 DNA (Ghosh and Singh 2018). In yeast Nsr1, RGG motifs also appear to play an important function. Yeast cells expressing Nsr1 with the truncation of this motif (Nsr1∆RGG) do not show the severe defects in the pre-rRNA processing and cell growth that are typical of the *nsr1∆* cells (Figure S3, A–C). However, when we deleted the RGG domain from Nsr1, the resulting Nsr1∆RGG construct lost the ability to complement the full-length Nsr1 in promoting genome instability at the *pTET-lys2-GTOP* cassette either in a *top1∆ nsr1∆* or *top1∆* background (Figure 3B). This could be due to weaker interaction between G4 DNA and the Nsr1∆RGG mutant protein. Earlier biochemical analysis showed that the RGG domain of hNCL is required for high-affinity interaction with G4-forming oligos *in vitro* (Hanakahi *et al.* 1999). Our ChIP results, showing that *in vivo* binding of Nsr1 to a G4 DNA-containing genomic locus is dependent on the presence of the RGG domain as well as the results of *in vitro* pull-down assay showing that binding to G4-forming oligo is significantly reduced when the RGG domain is absent, further support this hypothesis (Figure 5 and Figure S4).

Overall, our data indicate that the elevation of G4-associated genomic instability by Nsr1 requires a strong physical interaction with G4 DNA as mediated by the RGG domain. Such a conclusion suggests that Nsr1 binding leads to stabilization of the cotranscriptionally formed G4 DNA and that the Nsr1-bound G4 DNA forms a nucleoprotein complex that can significantly impede replication. Nonhistone protein–DNA complexes forming a replication block have been previously reported for the origin of replication (ORC) complex and Rap1 (Ivessa *et al.* 2003). To determine whether the Nsr1–G4 DNA complex poses a physical replication obstruction, we measured the replication kinetics of genomic region proximal to the G4 DNA-containing *pTET-lys2-GTOP* cassette using the ddPCR approach, where the copy number of the specific genomic loci was tracked through a single synchronized S phase. As shown in Figure 6, replication proceeds from *ARS306* through “KanMX” regions and the *pTET-lys2-GTOP* cassette before it passes the “STE50” locus. As would be expected from the replication block forming at the *pTET-lys2-GTOP*, no significant deviation in replication kinetics was observed at the sites located between the replication origin and the *pTET-lys2-GTOP* (*i.e.*, “ARS306” and “KanMX”; Figure 6, B and C). However, the replication fork must proceed past the G4-containing *pTET-lys2-GTOP* region before it reaches “STE50”, where the significant delay in replication was observed in the presence of Nsr1 in a *top1∆* background (Figure 6D). This delay in replication progress, just as the elevated G4-associated recombination, required the RGG domain of Nsr1; replication at “STE50” proceeded with significantly faster kinetics in cells expressing Nsr1∆RGG compared to cells expressing the full-length Nsr1. This difference between Nsr1∆RGG and Nsr1 further supports the idea that the strong physical interaction between Nsr1 and G4 DNA underlies both G4-specific replication obstruction and genome instability. Replication obstruction incurred by the combination of Nsr1 and G4 DNA is more clearly illustrated when the replication kinetics in cells containing cassette is compared to those in cells containing the non-G4-forming *pTET-lys2-GBTM* cassette as shown in Figure 7. A very significant delay in the replication progress is observed at the “STE50” locus downstream of the G4 reporter only in cells expressing full-length Nsr1. In *nsr1∆RGG* backgrounds, the replication kinetics at this locus showed no significant differences between the strains containing either the *pTET-lys2-GTOP* or *-GBTM* construct.

In summary, we have identified a novel function of yeast Nsr1 as a G4 DNA-binding protein. We first demonstrate here that Nsr1 is specifically enriched at a cotranscriptionally formed G4 DNA *in vivo* and that Nsr1-interaction with G4 DNA results in a significant replication impediment in a G4 DNA-specific manner. This key finding suggests that the formation of a stable Nsr1–G4 DNA complex functioning as a replication obstruction underlies the significant elevation in G4-associated genome instability. Importantly, data presented here point to the requirement of the conserved RGG domain at the C-terminal end of Nsr1 in promoting instability at G4 DNA. This result calls for further study into the conformational changes associated with the RGG domain in the Nsr1–G4 DNA complex that could enable such a complex to function as a replication obstacle.

### Summary

Here, we report a novel finding that the conserved G4 DNA-binding protein Nsr1 elevates recombination and chromosomal rearrangement occurring at a G4 DNA-forming sequence in the yeast genome. Elevated instability requires the C-terminally located RGG domain of Nsr1. Connection between genome instability and the function of Nsr1 to form a stable complex with G4 DNA led to the hypothesis that the Nsr1–G4 DNA complexes impede replication. We demonstrate that the presence of Nsr1 in fact slows replication past a G4 DNA-containing site and that the RGG domain is required to facilitate such a replication impediment.

## References

[bib1] Azevedo C., Livermore T., and Saiardi A., 2015 Protein polyphosphorylation of lysine residues by inorganic polyphosphate. Mol. Cell 58: 71–82. 10.1016/j.molcel.2015.02.010 25773596

[bib2] Bacolla A., Tainer J. A., Vasquez K. M., and Cooper D. N., 2016 Translocation and deletion breakpoints in cancer genomes are associated with potential non-B DNA-forming sequences. Nucleic Acids Res. 44: 5673–5688. 10.1093/nar/gkw261 27084947 PMC4937311

[bib3] Batrakou D. G., Heron E. D., and Nieduszynski C. A., 2018 Rapid high-resolution measurement of DNA replication timing by droplet digital PCR. Nucleic Acids Res. 46: e112 10.1093/nar/gky590 29986073 PMC6212846

[bib4] Berger C. M., Gaume X., and Bouvet P., 2015 The roles of nucleolin subcellular localization in cancer. Biochimie 113: 78–85. 10.1016/j.biochi.2015.03.023 25866190

[bib5] Bhattacharjee A., Wang Y., Diao J., and Price C. M., 2017 Dynamic DNA binding, junction recognition and G4 melting activity underlie the telomeric and genome-wide roles of human CST. Nucleic Acids Res. 45: 12311–12324. 10.1093/nar/gkx878 29040642 PMC5716219

[bib6] Bochman M. L., Paeschke K., and Zakian V. A., 2012 DNA secondary structures: stability and function of G-quadruplex structures. Nat. Rev. Genet. 13: 770–780. 10.1038/nrg3296 23032257 PMC3725559

[bib7] Brázda V., Haronikova L., Liao J. C., and Fojta M., 2014 DNA and RNA quadruplex-binding proteins. Int. J. Mol. Sci. 15: 17493–17517. 10.3390/ijms151017493 25268620 PMC4227175

[bib8] Cantor S. B., Bell D. W., Ganesan S., Kass E. M., Drapkin R., 2001 BACH1, a novel helicase-like protein, interacts directly with BRCA1 and contributes to its DNA repair function. Cell 105: 149–160. 10.1016/S0092-8674(01)00304-X 11301010

[bib9] Capra J. A., Paeschke K., Singh M., and Zakian V. A., 2010 G-quadruplex DNA sequences are evolutionarily conserved and associated with distinct genomic features in Saccharomyces cerevisiae. PLOS Comput. Biol. 6: e1000861 10.1371/journal.pcbi.1000861 20676380 PMC2908698

[bib10] Chen C., and Kolodner R. D., 1999 Gross chromosomal rearrangements in Saccharomyces cerevisiae replication and recombination defective mutants. Nat. Genet. 23: 81–85. 10.1038/12687 10471504

[bib11] Cogoi S., Shchekotikhin A. E., and Xodo L. E., 2014 HRAS is silenced by two neighboring G-quadruplexes and activated by MAZ, a zinc-finger transcription factor with DNA unfolding property. Nucleic Acids Res. 42: 8379–8388. 10.1093/nar/gku574 25013182 PMC4117790

[bib12] Dempsey L. A., Sun H., Hanakahi L. A., and Maizels N., 1999 G4 DNA binding by LR1 and its subunits, nucleolin and hnRNP D, A role for G-G pairing in immunoglobulin switch recombination. J. Biol. Chem. 274: 1066–1071. 10.1074/jbc.274.2.1066 9873052

[bib13] Du Z., Zhao Y., and Li N., 2008 Genome-wide analysis reveals regulatory role of G4 DNA in gene transcription. Genome Res. 18: 233–241. 10.1101/gr.6905408 18096746 PMC2203621

[bib14] Eddy J., and Maizels N., 2008 Conserved elements with potential to form polymorphic G-quadruplex structures in the first intron of human genes. Nucleic Acids Res. 36: 1321–1333. 10.1093/nar/gkm1138 18187510 PMC2275096

[bib15] Erard M. S., Belenguer P., Caizergues-Ferrer M., Pantaloni A., and Amalric F., 1988 A major nucleolar protein, nucleolin, induces chromatin decondensation by binding to histone H1. Eur. J. Biochem. 175: 525–530. 10.1111/j.1432-1033.1988.tb14224.x 3409881

[bib16] Fry M., 2007 Tetraplex DNA and its interacting proteins. Front. Biosci. 12: 4336–4351. 10.2741/2391 17485378

[bib17] Gallo A., Lo Sterzo C., Mori M., Di Matteo A., Bertini I., 2012 Structure of nucleophosmin DNA-binding domain and analysis of its complex with a G-quadruplex sequence from the c-MYC promoter. J. Biol. Chem. 287: 26539–26548. 10.1074/jbc.M112.371013 22707729 PMC3410995

[bib18] Ghosh M., and Singh M., 2018 RGG-box in hnRNPA1 specifically recognizes the telomere G-quadruplex DNA and enhances the G-quadruplex unfolding ability of UP1 domain. Nucleic Acids Res. 46: 10246–10261. 10.1093/nar/gky854 30247678 PMC6212785

[bib19] González V., and Hurley L. H., 2010 The C-terminus of nucleolin promotes the formation of the c-MYC G-quadruplex and inhibits c-MYC promoter activity. Biochemistry 49: 9706–9714. 10.1021/bi100509s 20932061 PMC2976822

[bib20] González V., Guo K., Hurley L., and Sun D., 2009 Identification and characterization of nucleolin as a c-myc G-quadruplex-binding protein. J. Biol. Chem. 284: 23622–23635. 10.1074/jbc.M109.018028 19581307 PMC2749137

[bib21] Haeusler A. R., Donnelly C. J., Periz G., Simko E. A., Shaw P. G., 2014 C9orf72 nucleotide repeat structures initiate molecular cascades of disease. Nature 507: 195–200. 10.1038/nature13124 24598541 PMC4046618

[bib22] Hanakahi L. A., Sun H., and Maizels N., 1999 High affinity interactions of nucleolin with G-G-paired rDNA. J. Biol. Chem. 274: 15908–15912. 10.1074/jbc.274.22.15908 10336496

[bib23] Hanakahi L. A., Bu Z., and Maizels N., 2000 The C-terminal domain of nucleolin accelerates nucleic acid annealing. Biochemistry 39: 15493–15499. 10.1021/bi001683y 11112535

[bib24] Han J., and van Hoof A., 2016 The RNA exosome channeling and direct access conformations have distinct in vivo functions. Cell Rep. 16: 3348–3358. 10.1016/j.celrep.2016.08.059 27653695 PMC5044803

[bib25] Hershman S. G., Chen Q., Lee J. Y., Kozak M. L., Yue P., 2008 Genomic distribution and functional analyses of potential G-quadruplex-forming sequences in Saccharomyces cerevisiae. Nucleic Acids Res. 36: 144–156. 10.1093/nar/gkm986 17999996 PMC2248735

[bib26] Huber M. D., Lee D. C., and Maizels N., 2002 G4 DNA unwinding by BLM and Sgs1p: substrate specificity and substrate-specific inhibition. Nucleic Acids Res. 30: 3954–3961. 10.1093/nar/gkf530 12235379 PMC137114

[bib27] Huppert J. L., and Balasubramanian S., 2005 Prevalence of quadruplexes in the human genome. Nucleic Acids Res. 33: 2908–2916. 10.1093/nar/gki609 15914667 PMC1140081

[bib28] Indig F. E., Rybanska I., Karmakar P., Devulapalli C., Fu H., 2012 Nucleolin inhibits G4 oligonucleotide unwinding by Werner helicase. PLoS One 7: e35229 10.1371/journal.pone.0035229 22675465 PMC3366963

[bib29] Ivessa A. S., Lenzmeier B. A., Bessler J. B., Goudsouzian L. K., Schnakenberg S. L., 2003 The Saccharomyces cerevisiae helicase Rrm3p facilitates replication past nonhistone protein-DNA complexes. Mol. Cell 12: 1525–1536. 10.1016/S1097-2765(03)00456-8 14690605

[bib30] Kim N., 2019 The interplay between G-quadruplex and transcription. Curr. Med. Chem. 26: 2898–2917. 10.2174/0929867325666171229132619 29284393 PMC6026074

[bib31] Kim N., and Jinks-Robertson S., 2011 Guanine repeat-containing sequences confer transcription-dependent instability in an orientation-specific manner in yeast. DNA Repair (Amst.) 10: 953–960. 10.1016/j.dnarep.2011.07.002 21813340 PMC3162091

[bib32] Kim N., Abdulovic A. L., Gealy R., Lippert M. J., and Jinks-Robertson S., 2007 Transcription-associated mutagenesis in yeast is directly proportional to the level of gene expression and influenced by the direction of DNA replication. DNA Repair (Amst.) 6: 1285–1296. 10.1016/j.dnarep.2007.02.023 17398168 PMC2034516

[bib33] Kondo K., and Inouye M., 1992 Yeast NSR1 protein that has structural similarity to mammalian nucleolin is involved in pre-rRNA processing. J. Biol. Chem. 267: 16252–16258. 1644811

[bib34] Lee W. C., Xue Z. X., and Melese T., 1991 The NSR1 gene encodes a protein that specifically binds nuclear localization sequences and has two RNA recognition motifs. J. Cell Biol. 113: 1–12. 10.1083/jcb.113.1.1 1706724 PMC2288927

[bib35] Lee W. C., Zabetakis D., and Melese T., 1992 NSR1 is required for pre-rRNA processing and for the proper maintenance of steady-state levels of ribosomal subunits. Mol. Cell. Biol. 12: 3865–3871. 10.1128/MCB.12.9.3865 1508189 PMC360260

[bib36] Li Q. J., Tong X. J., Duan Y. M., and Zhou J. Q., 2013 Characterization of the intramolecular G-quadruplex promoting activity of Est1. FEBS Lett. 587: 659–665. 10.1016/j.febslet.2013.01.024 23376615

[bib37] Lopes J., Piazza A., Bermejo R., Kriegsman B., Colosio A., 2011 G-quadruplex-induced instability during leading-strand replication. EMBO J. 30: 4033–4046. 10.1038/emboj.2011.316 21873979 PMC3209785

[bib38] Lopez C. R., Singh S., Hambarde S., Griffin W. C., Gao J., 2017 Yeast Sub1 and human PC4 are G-quadruplex binding proteins that suppress genome instability at co-transcriptionally formed G4 DNA. Nucleic Acids Res. 45: 5850–5862. 10.1093/nar/gkx201 28369605 PMC5449603

[bib39] Maizels N., 2015 G4-associated human diseases. EMBO Rep. 16: 910–922. 10.15252/embr.201540607 26150098 PMC4552485

[bib40] Maizels N., and Gray L. T., 2013 The G4 genome. PLoS Genet. 9: e1003468 10.1371/journal.pgen.1003468 23637633 PMC3630100

[bib41] Majounie E., Abramzon Y., Renton A. E., Perry R., Bassett S. S., 2012 Repeat expansion in C9ORF72 in Alzheimer’s disease. N. Engl. J. Med. 366: 283–284. 10.1056/NEJMc1113592 22216764 PMC3513272

[bib42] Megonigal M. D., Fertala J., and Bjornsti M. A., 1997 Alterations in the catalytic activity of yeast DNA topoisomerase I result in cell cycle arrest and cell death. J. Biol. Chem. 272: 12801–12808. 10.1074/jbc.272.19.12801 9139740

[bib43] Mendoza O., Bourdoncle A., Boule J. B., Brosh R. M. Jr., and Mergny J. L., 2016 G-quadruplexes and helicases. Nucleic Acids Res. 44: 1989–2006. 10.1093/nar/gkw079 26883636 PMC4797304

[bib44] Moruno-Manchon J. F., Koellhoffer E. C., Gopakumar J., Hambarde S., Kim N., 2017 The G-quadruplex DNA stabilizing drug pyridostatin promotes DNA damage and downregulates transcription of Brca1 in neurons. Aging (Albany NY) 9: 1957–1970. 10.18632/aging.101282 28904242 PMC5636668

[bib45] Otake Y., Soundararajan S., Sengupta T. K., Kio E. A., Smith J. C., 2007 Overexpression of nucleolin in chronic lymphocytic leukemia cells induces stabilization of bcl2 mRNA. Blood 109: 3069–3075. 10.1182/blood-2006-08-043257 17179226 PMC1852223

[bib46] Owiti N., Wei S., Bhagwat A. S., and Kim N., 2018 Unscheduled DNA synthesis leads to elevated uracil residues at highly transcribed genomic loci in Saccharomyces cerevisiae. PLoS Genet. 14: e1007516 10.1371/journal.pgen.1007516 30016327 PMC6063437

[bib47] Paeschke K., Bochman M. L., Garcia P. D., Cejka P., Friedman K. L., 2013 Pif1 family helicases suppress genome instability at G-quadruplex motifs. Nature 497: 458–462. 10.1038/nature12149 23657261 PMC3680789

[bib48] Pedroso I. M., Hayward W., and Fletcher T. M., 2009 The effect of the TRF2 N-terminal and TRFH regions on telomeric G-quadruplex structures. Nucleic Acids Res. 37: 1541–1554. 10.1093/nar/gkn1081 19139067 PMC2655686

[bib49] Peled J. U., Kuang F. L., Iglesias-Ussel M. D., Roa S., Kalis S. L., 2008 The biochemistry of somatic hypermutation. Annu. Rev. Immunol. 26: 481–511. 10.1146/annurev.immunol.26.021607.090236 18304001

[bib50] Piazza A., Serero A., Boule J. B., Legoix-Ne P., Lopes J., 2012 Stimulation of gross chromosomal rearrangements by the human CEB1 and CEB25 minisatellites in Saccharomyces cerevisiae depends on G-quadruplexes or Cdc13. PLoS Genet. 8: e1003033 10.1371/journal.pgen.1003033 23133402 PMC3486850

[bib51] Pinheiro L. B., Coleman V. A., Hindson C. M., Herrmann J., Hindson B. J., 2012 Evaluation of a droplet digital polymerase chain reaction format for DNA copy number quantification. Anal. Chem. 84: 1003–1011. 10.1021/ac202578x 22122760 PMC3260738

[bib52] Rawal P., Kummarasetti V. B., Ravindran J., Kumar N., Halder K., 2006 Genome-wide prediction of G4 DNA as regulatory motifs: role in Escherichia coli global regulation. Genome Res. 16: 644–655. 10.1101/gr.4508806 16651665 PMC1457047

[bib53] Sarkies P., Reams C., Simpson L. J., and Sale J. E., 2010 Epigenetic instability due to defective replication of structured DNA. Mol. Cell 40: 703–713. 10.1016/j.molcel.2010.11.009 21145480 PMC3145961

[bib54] Satake Y., Kuwano Y., Nishikawa T., Fujita K., Saijo S., 2018 Nucleolin facilitates nuclear retention of an ultraconserved region containing TRA2beta4 and accelerates colon cancer cell growth. Oncotarget 9: 26817–26833. 10.18632/oncotarget.25510 29928487 PMC6003563

[bib55] Seal S., Thompson D., Renwick A., Elliott A., Kelly P., 2006 Truncating mutations in the Fanconi anemia J gene BRIP1 are low-penetrance breast cancer susceptibility alleles. Nat. Genet. 38: 1239–1241. 10.1038/ng1902 17033622

[bib56] Seenisamy J., Rezler E. M., Powell T. J., Tye D., Gokhale V., 2004 The dynamic character of the G-quadruplex element in the c-MYC promoter and modification by TMPyP4. J. Am. Chem. Soc. 126: 8702–8709. 10.1021/ja040022b 15250722

[bib57] Siddiqui-Jain A., Grand C. L., Bearss D. J., and Hurley L. H., 2002 Direct evidence for a G-quadruplex in a promoter region and its targeting with a small molecule to repress c-MYC transcription. Proc. Natl. Acad. Sci. USA 99: 11593–11598. 10.1073/pnas.182256799 12195017 PMC129314

[bib58] Spell R. M., and Jinks-Robertson S., 2004 Determination of mitotic recombination rates by fluctuation analysis in Saccharomyces cerevisiae. Methods Mol. Biol. 262: 3–12. 10.1385/1-59259-761-0:003 14769952

[bib59] Sun H., Karow J. K., Hickson I. D., and Maizels N., 1998 The Bloom’s syndrome helicase unwinds G4 DNA. J. Biol. Chem. 273: 27587–27592. 10.1074/jbc.273.42.27587 9765292

[bib60] Tajrishi M. M., Tuteja R., and Tuteja N., 2011 Nucleolin: the most abundant multifunctional phosphoprotein of nucleolus. Commun. Integr. Biol. 4: 267–275. 10.4161/cib.4.3.14884 21980556 PMC3187884

[bib61] Thandapani P., O’Connor T. R., Bailey T. L., and Richard S., 2013 Defining the RGG/RG motif. Mol. Cell 50: 613–623. 10.1016/j.molcel.2013.05.021 23746349

[bib62] Todd A. K., Johnston M., and Neidle S., 2005 Highly prevalent putative quadruplex sequence motifs in human DNA. Nucleic Acids Res. 33: 2901–2907. 10.1093/nar/gki553 15914666 PMC1140077

[bib63] Tosoni E., Frasson I., Scalabrin M., Perrone R., Butovskaya E., 2015 Nucleolin stabilizes G-quadruplex structures folded by the LTR promoter and silences HIV-1 viral transcription. Nucleic Acids Res. 43: 8884–8897. 10.1093/nar/gkv897 26354862 PMC4605322

[bib64] Wahba L., Amon J. D., Koshland D., and Vuica-Ross M., 2011 RNase H and multiple RNA biogenesis factors cooperate to prevent RNA:DNA hybrids from generating genome instability. Mol. Cell 44: 978–988. 10.1016/j.molcel.2011.10.017 22195970 PMC3271842

[bib65] Williams J. D., Fleetwood S., Berroyer A., Kim N., and Larson E. D., 2015 Sites of instability in the human TCF3 (E2A) gene adopt G-quadruplex DNA structures in vitro. Front. Genet. 6: 177 10.3389/fgene.2015.00177 26029241 PMC4426816

[bib66] Williams J. D., Houserova D., Johnson B. R., Dyniewski B., Berroyer A., 2020 Characterization of long G4-rich enhancer-associated genomic regions engaging in a novel loop:loop ’G4 Kissing’ interaction. Nucleic Acids Res. 48: 5907–5925. 10.1093/nar/gkaa357 32383760 PMC7293029

[bib67] Wu Y., Shin-ya K., and Brosh R. M. Jr, 2008 FANCJ helicase defective in Fanconia anemia and breast cancer unwinds G-quadruplex DNA to defend genomic stability. Mol. Cell. Biol. 28: 4116–4128. 10.1128/MCB.02210-07 18426915 PMC2423121

[bib68] Yadav P., Harcy V., Argueso J. L., Dominska M., Jinks-Robertson S., 2014 Topoisomerase I plays a critical role in suppressing genome instability at a highly transcribed G-quadruplex-forming sequence. PLoS Genet. 10: e1004839 10.1371/journal.pgen.1004839 25473964 PMC4256205

[bib69] Yadav P., Owiti N., and Kim N., 2016 The role of topoisomerase I in suppressing genome instability associated with a highly transcribed guanine-rich sequence is not restricted to preventing RNA:DNA hybrid accumulation. Nucleic Acids Res. 44: 718–729. 10.1093/nar/gkv1152 26527723 PMC4737143

